# Application of Staining Methods to Compare Chromatin Condensation in Fresh and Freeze‐Thawed Dog Semen

**DOI:** 10.1111/rda.70023

**Published:** 2025-02-28

**Authors:** Elisa‐Marie Laux, Abbas Farshad, Axel Wehrend, Mohamad Eid Hammadeh

**Affiliations:** ^1^ Veterinary Clinic for Reproductive Medicine and Neonatology Justus‐Liebig‐University of Giessen Giessen Germany; ^2^ Obstetrics & Gynecology, Department of Biochemistry and Molecular Biology of Reproductive Medicine Laboratory University of Saarlandes Hombur/Saar Germany

**Keywords:** canine, chromatin, condensation, cryopreservation, sperm

## Abstract

Various staining techniques have been used for canine sperm analysis, but direct comparisons using identical semen samples are lacking. This study aimed to assess the efficiency, time requirements and cost‐effectiveness of different staining techniques: aniline blue, toluidine blue, acridine orange (AcO), chromomycin A3 (CMA3) and terminal deoxynucleotidyl transferase d‐UTP nick‐end labeling (TUNEL). Forty semen samples (20 fresh and 20 frozen–thawed) were used to assess chromatin condensation. Significant differences (*p* < 0.01) were found using two‐factor repeated measures variance analysis. Aniline blue staining differed significantly (*p* < 0.01) from toluidine blue, AcO and CMA3 staining. A significant difference (*p* < 0.05) was observed between AcO and TUNEL staining for fresh sperm, with no significant differences between TUNEL and other methods. Correlations in fresh sperm samples showed *r* = 0.567 between AcO and aniline blue, *r* = 0.645 between AcO and CMA3, and correlations of 0.455 and 0.557 for aniline blue – toluidine blue and aniline blue – TUNEL, respectively. For frozen samples, significant differences were found between aniline blue and toluidine blue and AcO tests (*p* < 0.05), and between CMA3 and TUNEL staining (*p* < 0.01). Correlations in frozen samples showed *r* = 0.582 between AcO and aniline blue, *r* = 0.752 between AcO and CMA3, *r* = 0.698 between toluidine blue and aniline blue, and *r* = 0.536 between CMA3 and aniline blue. A minimal association was found between standard semen analysis and chromatin analysis. In conclusion, toluidine blue is effective for light microscopy staining, while CMA3 is recommended for fluorescence microscopy due to its simplicity, rapidity and cost‐effectiveness.

## 1 Introduction

The assessment of sperm cell morphology is a critical element in the field of spermatology (Root Kustritz 2007). Abnormalities in sperm can be classified into two main categories: primary defects, which occur during spermatogenesis, and secondary defects, which develop during the maturation process. These abnormalities can be further distinguished based on their specific locations, such as acrosomal, head, midpiece and tail defects (Seager and Platz 1977). Research has demonstrated that the percentage of normal sperm is a significant predictor of litter size and pregnancy outcomes (Hollinshead et al. 2020). Furthermore, the selection of an appropriate staining technique is essential for sperm analysis, irrespective of the method employed, as it significantly influences the accuracy of the results (Freneau et al. 2010). It is imperative that the chosen staining method maintains the physiological integrity of the spermatozoa while effectively highlighting the majority of their morphological characteristics (Maree et al. 2010). Therefore, a range of staining methods is employed, each with distinct technical requirements and costs, to analyse these structures. In this context, Surmac et al. (2022) compared two sperm staining systems, Sperm Blue and Sperm Stain, for morphological analysis in canine sperm. Other researchers have suggested that chromatin staining is more frequently used for other animal species than for canines (Chohan et al. 2006; Erenpreiss et al. 2001; López‐Fernández et al. 2008; Love 2005; Mickelsen et al. 1993; Monachesi et al. 2019, 2022; Oleszczuk et al. 2016; Prinosilova et al. 2012; Rijsselaere et al. 2007; Rota et al. 2005; Sakkas et al. 2003; Zeqiraj et al. 2018).

Some studies have reported the use of light microscopy within this specific context (Dadoune et al. 1988; Terquem and Dadoune 1983). Additionally, fluorescence microscopy has also been utilised (Erenpreiss et al. 2004; Mello 1982). For instance, aniline blue staining rapidly distinguishes between pre‐laminated sperm, which remains uncoloured due to fully condensed chromatin, and well‐condensed sperm, which stains light blue (Terquem and Dadoune 1983). Sperms with inadequate chromatin condensation are recognised by their dark blue staining with aniline blue (Terquem and Dadoune 1983; Bianchi et al. 1993). Toluidine blue staining, which is a light microscopic staining method, has been used to determine chromatin condensation in sperms. Well‐condensed sperms stain light blue, whereas immature sperms stain violet (Erenpreiss et al. 2004). Chromomycin A3 (CMA3), another fluorescent dye, attaches to guanine/cytosine‐rich areas of DNA and competes with protamines for binding sites. This leads to the fluorescent staining of condensed chromatin, which has been used in the field of human medicine (Bianchi et al. 1996; Kazerooni et al. 2009; Manochantr et al. 2012). CMA3 staining has been reliably used to detect protamine deficiency in bovine sperm cells (Simões et al. 2009). Lower proportions of CMA3‐positive sperms in rabbits, pigs, goats, sheep, rats and rhesus monkeys than in humans have been reported (Warntjen 2012). However, research on the CMA3 staining patterns of canine sperms is lacking. Finally, acridine orange (AcO) is a fluorescent dye that binds to single‐stranded DNA as a polymer, resulting in red fluorescence. Conversely, it interacts with double‐stranded DNA in a monomeric state, producing green fluorescence. This property is used to assess chromatin condensation both cytometrically and with fluorescence microscopy (Kosower et al. 1992). The terminal deoxynucleotidyl transferase d‐UTP nick‐end labeling (TUNEL) staining technique, which was originally developed by Gorczyca et al. (1993) to detect apoptosis in human sperms, utilises fluorescently labelled nucleotides (d‐UTP) to detect DNA strand breaks. These breaks, observable under fluorescent microscopy, are directly associated with the exposed OH' ends and are widely used in contemporary fertility research (Chohan et al. 2006; Lopes et al. 1998).

On the other hand, Strotmann (2009) highlights the wide variation in sperm chromatin stability in male dogs, attributing this to the lack of fertility‐based selection in breeding. This suggests the need for detailed assessment methods for canine spermatozoa to aid in selective breeding. Evidence of a correlation between chromatin status and fertility in humans and other species motivates the introduction of chromatin condensation studies in dogs. Recent studies have shown varied results regarding the correlation between conventional semen parameters and fertility in dogs (Mickelsen et al. 1993; Choi et al. 2011; Prinosilova et al. 2012). In this context, various staining techniques have been documented in canine spermatology, some of which have been used for decades. Furthermore, research on chromatin condensation in male dogs is sparse, resulting in a notable diagnostic discrepancy compared to human studies. Finally, in human medicine, the assessment of sperm chromatin has proven to be an important technique in spermatological diagnostics Strotmann (2009), which was used in veterinary medicine more extensively for other animal species in the past, suggesting that the introduction of similar approaches for male dogs would be beneficial. Consequently, the main aim of this research was therefore to improve andrological diagnostics in male dogs by integrating spermatological staining techniques that are not yet widely used in small animal veterinary practice. These techniques are already well validated in human medicine and have been used in species other than dogs. In particular, this study aims to compare the efficacy of aniline blue and toluidine blue staining as methods of light microscopy with AcO, CMA3 and TUNEL staining as methods of fluorescence microscopy. We also want to evaluate the time efficiency and cost efficiency of these staining methods.

## 2 Materials and Methods

### 2.1 Chemicals and Ethical Approval

All chemicals used were provided by Morphisto GmbH (Offenbach am Main, Germany), Sigma‐Aldrich (Darmstadt, Germany), Roche Molecular Biochemicals (Penzberg, Germany) and Cell Technology Company (Danvers, Massachusetts, USA), unless otherwise indicated.

Experimental procedures were performed according to the regulations established by the Justus‐Liebig University of Giessen, Giessen, Germany, to ensure the welfare of the experimental animals. The use of the sperm samples was approved by the local ethics authority with the mediation of the Animal Welfare Office of Justus‐Liebig College Giessen, which is confirmed by internal documents and the assigned IRB number kTV 11‐2018.

### 2.2 Semen, Preparation and Evaluation

The study involved the collection and analysis of 40 dog semen samples, comprising 20 fresh and 20 frozen–thawed. These samples were obtained from healthy and fertile dogs of different ages (proximately between 3.5 and 7 years) and breeds provided by private homes for andrological assessment at the Veterinary Clinic for Reproductive Medicine and Neonatology of the Justus Liebig University of Giessen, Germany (Table 1).

**TABLE 1:** Descriptions of 40 different canine breeds, including 20 breeds utilised for the analysis of fresh semen and 20 breeds for the investigation of thawed semen.

Used dogs per breeds	Native semen	Used dogs per breeds	Frozen–thawed semen
4	Yorkshire Terriers	11	unknown
2	Leonbergers	1	Rottweiler
3	German Shepherds	1	Groenendael
1	Gordon Setter	1	Bullmastiff
1	Border Collie	2	Bullterriers
1	Giant Schnauzer	1	Boxer
2	Great Danes	1	mixed breed
1	Rottweiler	2	German Shepherd
2	Rhodesian Ridgeback	—	—
1	Greater Swiss Mountain Dog	—	—
2	Mixed‐breed dogs	—	—

Semen was manually obtained through stimulation to ensure the collection of different parts of the ejaculate. Each ejaculate was individually assessed for pre‐sectional, sperm‐rich and post‐secretion fractions (Hermansson and Linde Forsberg 2006). These specific fractions were carefully collected in tulip‐shaped glasses preheated to 37°C. Only the sperm‐rich second fraction was used. Semen was transported to the lab and kept at 37°C. Samples with > 70%–75% progressive motility and normal morphology were selected for cryopreservation. Samples were diluted with a tris‐fructose egg yolk diluent to a final concentration of 70–60 × 10^6^ sperm/mL, filled into 0.5 mL straws, cooled to 5°C for 1.5 h and, then, frozen in nitrogen vapour for 10 min before storage in liquid nitrogen. Each straw was thawed at 37°C for 1 min.

The preparation of spray‐fixed smears followed a specific procedure, which included preparing smears and fixing them for microscopic staining. To enhance the precision of the evaluation, a 1:1 dilution of the sperm‐rich fraction with HAM's F10 solution was carried out. Subsequently, 5 μL of the sperm suspension was applied to a second slide. After 10 min of air drying, the slides were fixed by bending them in the appropriate direction and applying two to three sprays of M‐Fix Fixierspray fixative solution. Smears were stored at room temperature in borate‐buffered saline until staining.

### 2.3 Staining Techniques

#### 2.3.1 Aniline Blue Staining for the Assessment of Sperm Chromatin Condensation

Following the method by Terquem and Dadoune (1983), modified for feline spermatozoa by Hingst et al. (1995), slides were fixed using a 3% glutaraldehyde solution applied by the spray method and soaked for 30 min, then air‐dried for 5 min. Slides were stained with a 5% aniline blue solution (pH 3.5) for 5 min, washed in PBS, air‐dried and coated with Entellan. Five hundred spermatozoa were counted using an Olympus BX41 light microscope (1000× magnification) and categorised as aniline blue‐positive or blue‐negative based on head colour, with dark blue indicating positivity. Aniline blue‐positive spermatozoa are those that stained dark blue in their heads, indicating chromatin condensation anomalies or DNA fragmentation. Aniline blue‐negative spermatozoa are those that did not stain dark blue, suggesting normal chromatin condensation.

#### 2.3.2 Toluidine Blue Staining Technique for the Assessment of Sperm Chromatin Instability

According to Erenpreiss et al. (2001), the toluidine blue staining procedure involved fixing the slides at 4°C for 30 min in a 1:1 mixture of 96% ethanol and acetone, followed by hydrolysis in 0.1 N HCl for 5 min, also at 4°C. Slides were then triple‐washed in distilled water for 2 min each. Samples were stained for 10 min in 0.05% toluidine blue (pH 3.5), then dehydrated twice for 3 min each at 37°C in butanol and xylene and covered with Entellan. Dark blue or violet‐stained sperm heads indicated reduced chromatin condensation, while light blue indicated well‐condensed chromatin.

#### 2.3.3 Chromomycin A3 Staining for the Assessment of Sperm Chromatin Instability

According to Bianchi et al. (1993), slides were fixed using Carnoy's solution (methanol and acetic acid, 3:1) and air‐dried for 2 h. Samples were stained with 100 μL CMA3 solution (25 mg/mL in PBS) and incubated in the dark for 20 min. Slides were washed in PBS and coated with glycerol after staining. Using a fluorescence microscope at 1000× magnification (Olympus BH2‐RFCA, Germany) equipped with a 490 nm excitation filter, 500 sperm cells were counted. Positively stained cells, which emitted light‐green fluorescence, indicated chromatin anomalies, while negatively stained cells appeared darker green, indicating normal chromatin.

Additionally, a positive control using deprotamination was conducted following the protocol by Bizzaro et al. (1998), which was already applied to bull spermatozoa by Simões et al. (2009). For this, the semen was incubated in a solution of 5 mM DTT and 0.1% Triton X‐100 in PBS for 15 min at 37°C prior to fixation. This process is intended to break the disulphide bonds between protamine chains and partially permeabilise the plasma membrane. After washing the samples twice, the fixation and staining process was carried out as described above. A positive control verifies the staining method by showing expected results, confirming chromatin condensation or decondensation.

#### 2.3.4 Acridine Orange Staining for Assessing Sperm DNA Integrity

Using the Tejada et al. (1984) method, slides were spray‐fixed and treated with Carnoy's solution (methanol/acetic acid 3:1) for 2 h, then air‐dried. The staining involved 0.84 g citric acid monohydrate in 40 mL water, 2.5 mL 0.3 M disodium hydrogen phosphate and 10 mL 0.01 g acridine orange (AcO) in water, all prepared in a darkened room. Slides were stained for 5 min, washed and examined microscopically. Sperm cells that appeared red, yellow or orange were considered positive (damaged), whereas those that appeared green were considered negative (intact).

#### 2.3.5 TUNEL for the Assessment of Sperm Chromatin Integrity

Following Watanabe et al. (2011), the TUNEL assay was used to detect DNA damage. Slides were fixed in 4% paraformaldehyde for 60 min and permeabilised in 0.1% Triton X‐100 and sodium citrate at 4°C. The TUNEL mixture (label solution and enzyme, 10:1) was applied, and slides were incubated overnight at 37°C in the dark. After staining, the slides were counterstained with 10 μL of a 0.1% DAPI solution and examined. Green‐fluorescent sperm were identified as TUNEL‐positive, indicating DNA damage. Sperm lacking green fluorescence were classified as TUNEL‐negative, indicating undamaged DNA. For the frozen samples, a positive control was included in each staining run. After fixation and permeabilisation, 1000 U/mL recombinant DNAse from Roche was applied. Although the manufacturer recommends 3 U/mL, Villani et al. (2010) showed that bovine and murine sperm chromatin exhibit increased DNA fragmentation only at higher concentrations, so 1000 U/mL was used. Strict separation during washing, drying and mounting was required to avoid contamination of samples with DNAse or negative controls with the enzyme.

### 2.4 Time Requirements and Costs for Staining

The feasibility criteria for veterinary medical practice were established. Time for smear preparation, including incubation, staining, drying and slide processing, was recorded. Costs per smear and for staining 20 slides were calculated, excluding slides, coverslips and pipette tips. The modified fluorescence microscopy assay was not possible on fixed preparations. Safety data sheets were reviewed for potential risks to staff and the environment. Feasibility comparisons were made based on the findings.

### 2.5 Statistical Analysis

Statistical analyses were performed using BMDP/Dynamic Release 8.1 software. The colorations were tested for normal distribution using a Q‐Q plot and residual analysis, which showed a right‐skewed distribution. To further analyse the data, a logarithmic transformation was applied after replacing zero values with 0.1. Residual analyses were performed using the R statistical software. One‐way analysis of variance with repeated measures was used to compare the colours of the stains and determine significant differences between the staining methods. The Student–Newman–Keuls test was utilised for pairwise comparisons to determine specific differences in the outcomes of the chromatin staining techniques employed. Finally, correlation analysis using Pearson's correlation coefficient was conducted on all pairs of data with logarithmic values.

## 3 Results

**FIGURE 1:**
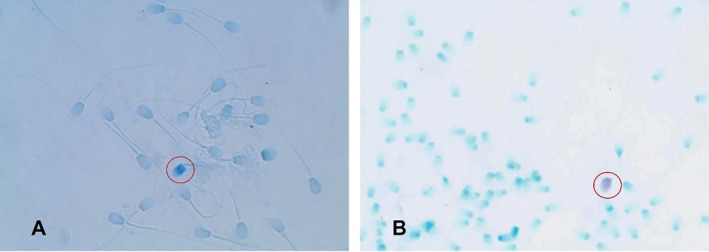
(A) Aniline blue staining of frozen dog sperm cells at 1000× magnification shows 23 sperm cells with one centrally stained spermatozoon. (B) Toluidine blue staining of native canine sperm at 1000× magnification (with immersion oil) reveals distinct metachromasia, highlighting the contrast between light blue‐negative and purple‐positive spermatozoa.

The aniline blue staining method indicates positivity through dark blue staining, ranging from 0% to 1.6%, with positives showing chromatin damage. Furthermore, the obtained results indicate that cryopreserved sperm had an average stained percentage of 0.75% ± 1.2%, while fresh semen samples averaged 0.33% ± 0.41%. This method is fast (21 s/slide) and economical, with a total prep time of 52 min. In this context, the toluidine blue staining showed positive results in fresh semen between 0% and 3.4%, and frozen ejaculates averaged 1.3%. This technique, simpler yet effective, yielded better‐stained spermatozoa, highlighted by dark purple‐positive cells. It is quicker (49 s/slide) but includes additional steps, totalling 72 min of prep and drying time. Costs are balanced by the reuse of butanol and xylene. Figure 1 illustrates sperm stained with aniline blue (A) and toluidine blue (B) highlighting the differences in staining patterns and indicating areas of damage.

**FIGURE 2:**
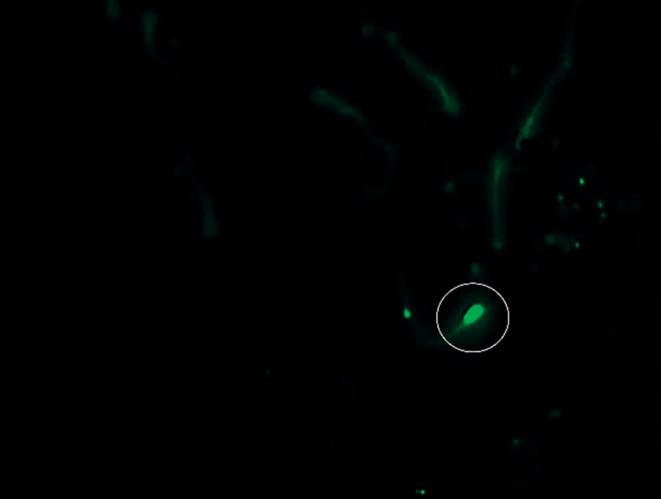
Chromomycin A3 staining of a fresh semen sample from a male dog at 1000× magnification reveals six non‐fluorescent sperm cells and one fluorescent sperm cell stained with CMA3.

**FIGURE 3:**
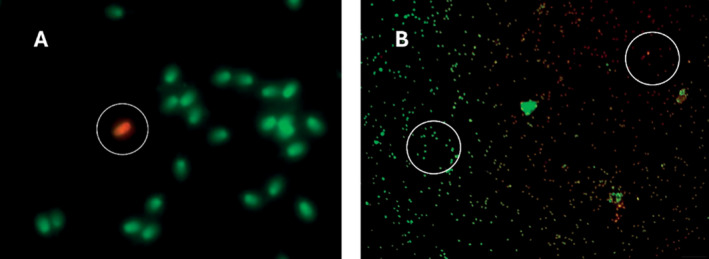
This figure indicates canine sperm samples treated with acridine orange. At 1000× magnification (A), a clear differentiation is observed: Green‐stained sperm cells indicate intact DNA, while red‐stained sperm cells signify damaged DNA. However, at 100× magnification (B), the challenges in evaluation due to uneven acridine orange staining become evident, with red‐stained sperm in the upper right quadrant and green‐stained sperm in the left half.

The assessment of CMA3 involved counting non‐staining mature sperm (Figure 2), with positive cells being readily distinguishable. The slides maintained their usability for several weeks at a temperature of −18°C. The results indicated that the peak percentage of CMA3‐positive sperm was 3.6% in fresh smears, while frozen–thawed samples exhibited a maximum of 8.0%. The overall average of CMA3‐positive sperm across all tested samples was found to be 1.13%. Specifically, the mean for fresh semen samples was recorded at 0.90%, which is lower than the overall average, while the cryopreserved samples yielded a mean value of 1.36%. CMA3 proved to be the most cost‐effective fluorescence technique, requiring just 21 s per slide and allowing other tasks during the 160‐min fixation and drying period. The effects of the AcO staining technique (Figure 3A,B) on sperm cells were observed. The staining process required 19 s per slide, culminating in a total preparation, incubation and drying time of 160 min. The evaluation of the staining results was complicated by the presence of inconsistent regions, which displayed both red‐ and green‐stained sperm areas. Utilising spray‐fixed smears proved to be both cost‐effective and time‐saving. The results from the AcO staining method showed that fresh sperm samples had a maximum percentage of 1.28% AcO‐positive sperm, whereas frozen samples achieved a maximum of 1.94%. The examination of twenty fresh semen samples indicated an average of 1.28% chromatin‐unstable sperm cells, while the mean value for cryopreserved samples was 1.94%. The results further show that although the highest recorded value for the fresh semen was 3.6%, the maximum value for frozen samples was 8.2%. In these contexts, Figures 2 and 3 depict sperm stained with CMA3 and AcO techniques, showcasing the differences in staining patterns and identifying areas of damage.

**FIGURE 4:**
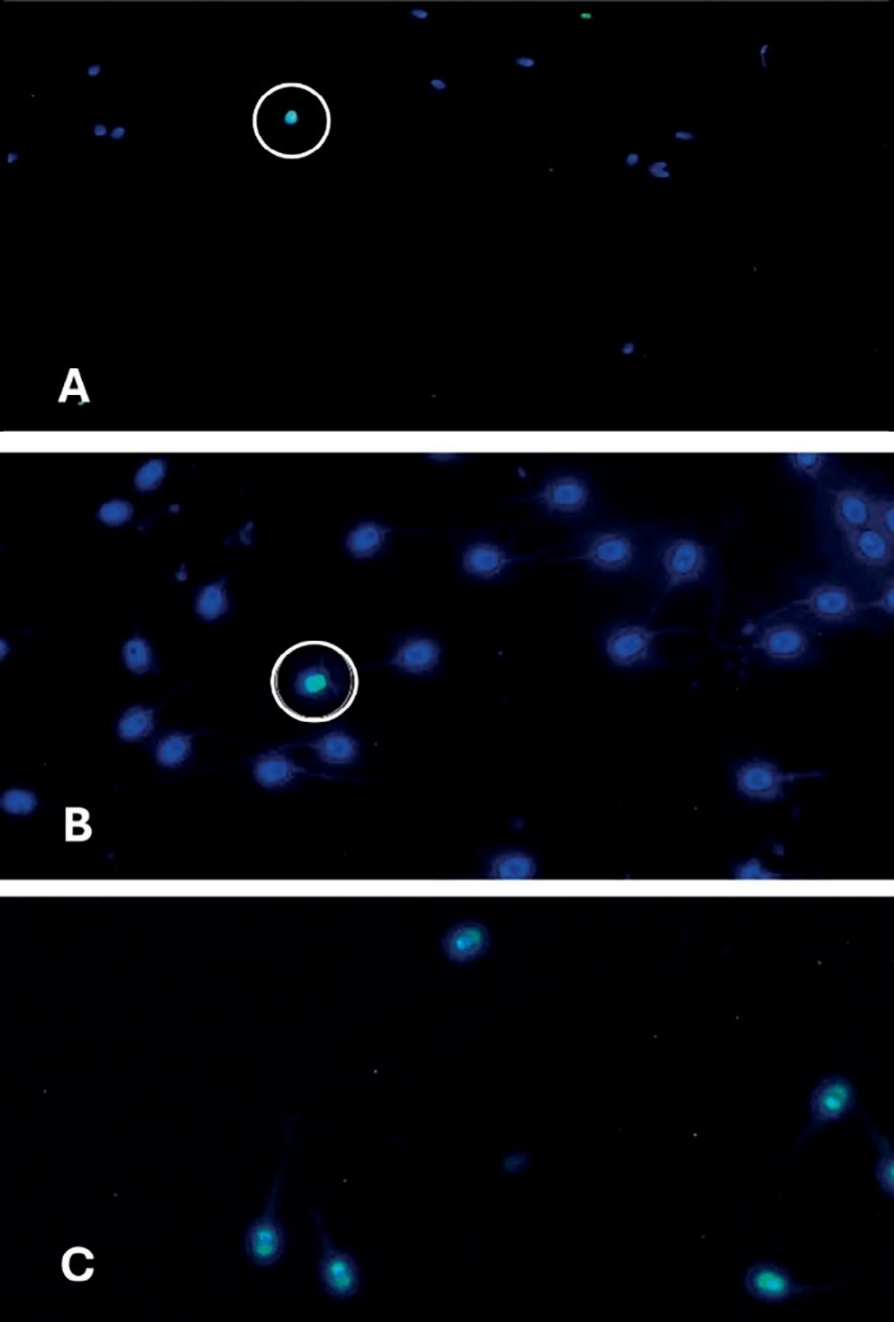
This figure shows a TUNEL‐stained frozen sperm smear from a male dog. In section A, 13 sperm cells are visible at 400× magnification, with DAPI staining (blue). One cell in the upper left green fluorescence, indicating DNA fragmentation. Section B shows TUNEL‐positive (green) and DAPI‐stained (blue) sperm cells without DNA fragmentation. Section C is a positive control using DNAse, showing green fluorescence for DNA fragmentation.

Beyond the overnight incubation step, the TUNEL test is quite straightforward. However, due to the requirement for DAPI counterstaining, it is necessary to capture and merge images, as TUNEL‐positive sperm are not easily discernible in the green FITC filter. Figure 4, specifically images A and B, display blue‐stained sperm that lack DNA fragmentation, while image C illustrates a positive control featuring green, fluorescent sperm. To mitigate fading, it is advisable to use short exposure times. Moreover, conducting sample preparation in a light‐protected setting is essential. The obtained data also show that, on average, fresh semen samples contained 0.95% TUNEL‐positive sperm, whereas the cryopreserved ejaculates averaged 1.42%. Additionally, both fresh and frozen–thawed samples, an average of 1.23% of sperm exhibited DNA decondensation. The maximum observed value for DNA‐damaged sperm reached 9.8%. Remarkably, one out of the twenty fresh semen samples showed no TUNEL‐positive cells when counting 500 sperm. Additionally, this method takes 30 s per slide plus 13 min for preparation. Although it is the most expensive staining method, it ensures precise results.

It is important to note that the one‐factor variance analysis revealed significant global differences in the comparison of all staining methods for both fresh and cryopreserved semen samples. This indicates that the staining methods are not equivalent and show statistically significant differences, meaning the results of one staining method cannot be generalised to another. A pairwise comparison of all group means using the Student–Newman–Keuls (Posthoc) method showed significant differences (*p* < 0.01) between the staining methods. Table 2 outlines the statistical significance between different staining methods for fresh semen, where aniline blue showed highly significant differences (*p* < 0.01) with Toluidine blue, AcO and CMA3, but no significant difference with TUNE. Toluidine blue showed highly significant differences (*p* < 0.01) with Aniline blue, but no significant differences with AcO, CMA3 and TUNEL. AcO showed highly significant differences (*p* < 0.01) with Aniline blue, no significant differences with Toluidine blue and CMA3 and significant differences (*p* < 0.05) with TUNEL. CMA3 showed highly significant differences (*p* < 0.01) with aniline blue, but no significant differences with Toluidine blue, AcO and TUNEL. TUNEL showed no significant differences with Aniline blue, Toluidine blue and CMA3, but significant differences (*p* < 0.05) with AcO. These results indicate that certain staining methods produce statistically different outcomes, affecting their comparability.

**TABLE 2:** Demonstration of significant differences (*p* < 0.01) between five different staining methods related to chromatin condensation in canine native semen.

	Aniline blue	Toluidine blue	AcO	CMA3	TUNEL
Aniline blue	x	++	++	++	−
Toluidine blue	++	x	−	−	−
AcO	++	−	x	−	+
CMA3	++	−	−	x	−
TUNEL	−	−	+	−	x

*Note:* Acridine Orange (AcO), Chromomycin A3 (CMA3), terminal deoxynucleotidyl transferase‐mediated d‐UTP nick‐end labeling (TUNEL), x: no comparison; −: no significant difference; +: significant difference with *p* < 0.05; ++: significant difference with *p* < 0.01.

Additionally, Table 3 highlights the statistical significance between staining methods for frozen–thawed sperm where aniline blue showed significant differences with Toluidine blue, AcO and CMA3 (*p* < 0.05), and highly significant differences with CMA3 and TUNEL (*p* < 0.01). Toluidine blue differed significantly from Aniline blue (*p* < 0.05). AcO showed significant differences with Aniline blue (*p* < 0.05) and TUNEL (*p* < 0.05). CMA3 showed highly significant differences with Aniline blue (*p* < 0.01). TUNEL was significantly different from AcO (*p* < 0.05). No significant differences were found between the other pairs.

**TABLE 3:** Demonstration of significant differences (*p* < 0.01) between five different staining methods related to chromatin condensation in canine cryopreserved semen.

	Aniline blue	Toluidine blue	AcO	CMA3	TUNEL
Aniline blue	x	+	+	++	++
Toluidine blue	+	x	−	−	−
AcO	+	−	x	−	−
CMA3	++	−	−	x	−
TUNEL	++	−	−	−	x

*Note:* Acridine Orange (AcO), ChromomycinA3 (CMA3), terminal deoxynucleotidyl transferase‐mediated d‐UTP nick‐end labeling (TUNEL), x: no comparison; −: no significant difference; +: significant difference with *p* < 0.05; ++: significant difference with *p* < 0.01.

Finally, Table 4 indicates a comparative analysis of the time and costs associated with five techniques for examining chromatin condensation in fresh and freeze‐thawed canine sperm.

**TABLE 4:** A comparative evaluation of the time and financial resources needed for five techniques utilised in the examination of chromatin condensation in native versus freeze‐thawed canine sperm.

Working time to prepare the slide samples						Costs of method per microscope slides		
Staining methods	Preparation time	Processing time (MS)	Pure incubation time (waiting)	Net working time	Total times/20 MS in min	Material per staining cycle (20 MS)	Material costs/MS in €	Total costs/MS^a^ in €
AB	MS‐Fixation: 5 min	Fixation: 3 s Remove from Fixation: 3 s Placing in staining solution: 3 s Remove from staining solution and wash: 12 s	Fixation: 25 min Drying: 10 min Staining: 7 min Draying: 10 min	21 s/MS 5 min prep. and waiting time	12 min working time and 52 min waiting time = Total: 64 min	200 mL of fixation solution 200 mL of 5% aniline blue Mounting medium	0.06 0.64 0.03	0.73
TB	Establish fixation: 3 min Prep. buffer: 5 min Hydrolysate solution prep.: 5 min Stain solution prep.: 3 min	Fixation: 3 s Immerse in hydrolysis solution: 3 s Remove: 3 s Immerse in staining solution: 3 s Washing the MS: 10 s Dehydration: 2 × 2 × 3 s Clarification: 2 × 2 × 3 s Cover: 5 s	Fixation: 30 min Drying: 10 min Hydrolyse: 5 min Drying: 10 min Staining: 5 min Dehydration: 2 × 3 min Clearing: 2 × 3 min	49 s/MS 16 min prep. and waiting time	34 min working time and 72 min waiting = Total: 106 min	200 mL Fixative solution 200 mL Hydrolysis solution 10 mg Toluidine blue 1.72 g Disodium hydrogen phosphate 2.92 g Citric acid monohydrate 400 mL Butanol 400 mL Xylol Mounting medium	0.48 0.07 0.01 0.01 0.01 0.6 0.5 0.03	**1.71**
CMA3	Prep. fixative solution: 3 min Prep. CMA3 working solution: 5 min	Place slide in fixative: 3 s Remove slide from fixative: 3 s Apply CMA3: 5 s Washing: 5 s Mount: 5 s	Fixation: 120 min Drying: 10 min Staining: 20 min Drying: 10 min	21 s/MS 8 min prep. and waiting time	15 min working time and 160 min waiting time = Total: 172 min	180 mL fixative solution 0.5 mg CMA3 2 mL PBS	0.09 0.48 0.01	**0.58**
AcO	Prep. stock solutions: 10 min Prep. fixative: 3 min	Place slide in fixative: 3 s Remove slide from fixative: 3 s Place slide in stain: 3 s Remove slide from stain and wash: 5 s Mount: 5 s	Fixation: 120 min Drying: 10 min Staining: 7 min	19 s/MS 13 min prep. and waiting time	19 min working time and 147 min waiting time = Total: 166 min	200 mL fixative solution 40 mg AcO 3.36 g citric acid monohydrate 1.32 g sodium hydrogen phosphate Mounting medium	0.09 0.01 0.01 0.01 0.01	**0.13**
TUNEL	Prep. permeabilisation solution: 5 min Prep. TUNEL mix: 3 min Prep. DAPI solution: 5 min	Place slide in fixative: 3 s Remove slide from fixative: 3 s Place slide in permeabilisation solution: 3 s Remove slide from permeabilisation solution and dipping: 6 s Apply TUNEL mix: 5 s Wash off TUNEL mix: 5 s Apply DAPI + mount: 5 s	Fixation: 120 min Permeabilisation: 10 min Incubation: overnight	30 s/MS 13 min prep. and waiting time	23 min working time 130 min waiting time overnight incubation = Total: 153 min and overnight incubation	In Situ Cell Death Kit, Fluorescein 0.2 mL Triton X‐100 0.025 mg DAPI 1000 U/mL DNase	5.64 0.01 0.01 0.16	**5.82**

Abbreviations: €, Euro; AB, Aniline Blue; AcO, Acridine Orange; CMA3, Chromomycine A3; Min., Minutes; MS, Microscope Slide; Prep, Preparation; s, Seconds; TB, Toluidine Blue; TUNEL, Terminal Deoxynucleotidyl transferase d‐UTP Nick‐end Labeling.

^a^ Total costs/MS without working costs. Bold value = Significant differ from other methods.

## 4 Discussion

Traditionally, researchers assessing male fertility have relied on standard semen analysis parameters (Monachesi et al. 2019, 2022). However, normal results do not always guarantee fertility (Prinosilova et al. 2012). In conventional veterinary practice, sperm morphology is typically assessed manually by examining a stained sample under an optical microscope. While this method may be prone to errors when performed by an inexperienced andrology technician, it remains the gold standard for evaluating canine sperm (Surmac et al. 2022). Consequently, this limitation has catalysed a shift towards the exploration of sperm nuclei and chromatin structure, which are critical determinants of sperm attributes and fertility success. In this context, modified fluorescence microscopy showed significant reproducibility (López‐Fernández et al. 2008; Oleszczuk et al. 2016; Rijsselaere et al. 2005; Zeqiraj et al. 2018). Various staining methods assessed chromatin condensation in human and animal sperm, with few studies on dogs. This study evaluated five staining methods to find a reliable technique for examining canine sperm. In the examination of positive staining results for sperm cells with chromatin abnormalities, it has been observed that fertile males generally present with < 20% of aniline blue‐positive spermatozoa, indicating a lower prevalence of chromatin abnormalities (Hammadeh et al. 1998). Conversely, the male dogs analysed in this study exhibited a significantly reduced incidence of aniline blue‐positive sperm, with an average of 0.51% ± 0.29%, suggesting an even lesser degree of chromatin irregularities. In comparison, other species, including tomcats, murine sperm and bulls, demonstrate a range of aniline blue‐positive sperm rates, highlighting species‐specific variations in sperm quality. For instance, tomcat sperm sourced from the cauda epididymis displayed an average of 92.2% ± 3.7% unstained heads (Hingst et al. 1995), while murine sperm positivity was recorded at < 1.88% (Warntjen 2012). In the investigation of bull sperm, Vieytes et al. (2008) identified a positivity range between 0% and 6.5%. Conversely, Khalifa et al. (2008) observed a wider range of 0% to 19% for aniline blue‐positive spermatozoa across six different bulls, with the mean percentage of the analysed ejaculates calculated at 11.22% ± 0.96%. Furthermore, in canine subjects, cryopreserved sperm demonstrated a higher rate of aniline blue positivity at 1.3%, compared to 0.92% in fresh sperm. Consequently, positive staining serves as a vital indicator of chromatin quality and integrity, which are essential for assessing sperm health across various species and conditions.

Our research demonstrated that the application of toluidine blue resulted in a 1.31% positivity rate for sperm, which is notably higher than the 0.51% achieved with aniline blue. The sample type had minimal effect on the staining results, indicating that toluidine blue is a suitable method for evaluating cryopreserved semen. In this context, our findings support those of Monachesi et al. (2019), who showed that toluidine blue effectively assesses sperm chromatin condensation and decondensation in raw canine semen, thereby enhancing the overall analysis of semen. Additionally, the literature review indicates that toluidine blue staining is effective in identifying positive sperm across several animal species. Furthermore, the findings of Beletti and Mello (2004) indicate that rabbits have a prevalence of 2.3%, whereas bulls demonstrate a prevalence that ranges from 0% to 0.06%. The values of 0.06%–0.68% have been reported for bovines (Mello 1982), which is consistent with the results of the current study. On the other hand, 24.9% has been reported for men (Chohan et al. 2006), while 7.55% has been reported for stallions (Carretero et al. 2012). The results of the current study demonstrated a higher percentage of stained sperm in frozen semen (1.3%) than in fresh semen (0.92%). This discrepancy is likely due to variations in the semen donors. There was no statistically significant interaction between the sample type and staining results. Therefore, the toluidine blue test was considered appropriate for frozen semen. Within this context, a study indicated higher toluidine blue‐positive sperm and more marked DNA fragmentation in fresh than in thawed semen (Carretero et al. 2012). Erenpreiss et al. (2004) highlights the unique properties of Toluidine Blue as an external dye, noting its ability to indicate interactions between DNA and proteins, thereby reflecting the compactness of chromatin. Additionally, it serves as a marker for DNA integrity. This assertion is supported by earlier studies (Erenpreisa et al. 1992, 1997), and Erenpreiss attributes a broader staining spectrum to Toluidine Blue compared to other staining techniques.

Mello (1982) documented a positive response of pathomorphous sperm derived from fertile bulls when treated with toluidine blue. Additionally, a notable correlation was identified between the presence of toluidine blue in bovine sperm and variations in the morphology and dimensions of the sperm head, while no significant relationship was found concerning changes in the sperm tail (Beletti and Mello 2004). The authors anticipated an association between chromatin condensation and head morphology considering the predominance of chromatin in the sperm head, as chromatin plays a crucial role in defining head structure and integrity. Despite the lack of a statistical association between sperm abnormalities and toluidine blue staining in the present study, the sample with the highest proportion of sperms with abnormal head morphology had the most positive outcomes for the toluidine blue test.

The results of this study indicate that canine semen shows significantly lower Chromomycin A3 (CMA3) values compared to the threshold for protamine deficiency in human spermatozoa, which was set at 31% by Zandemami et al. (2012). This difference between humans and animals was previously described by Bianchi et al. (1993), who stained human and murine spermatozoa with CMA3 before and after artificial protamination, demonstrating the correlation of CMA3 staining with inadequate protamination. They found that untreated samples from fertile men had between 15% and 20% CMA3‐positive spermatozoa, while mice showed < 1%. Warntjen (2012) found an average of 1.19% CMA3‐positive sperm in mice. Additionally, Warntjen (2012) investigated the protamination of other species, examining semen samples from rabbits, pigs, goats, sheep, rats and rhesus monkeys, all of which had significantly lower CMA3‐positive sperm percentages than humans. Human spermatozoa had a mean value of 58.77%, rhesus monkeys 1.35%, pigs 0.52%, sheep 0.24%, goats 0.13%, rabbits 0.85% and rats 0.19%. Overall, the CMA3 values in animals were much lower than in humans and can be compared to the findings in dogs. In bulls, CMA3 values are also in a similar range as in dogs, as determined by Simões et al. (2009) through the examination of thawed deep‐freeze samples of bovine spermatozoa. Out of fourteen bulls examined, only two showed elevated values of 0.2% ± 0.06% and 0.2% ± 0.0% positive sperm, while the remaining samples were below 0.1% ± 0.05%. It is important to emphasise that the staining patterns differ considerably between species, resulting in fewer CMA3‐positive sperm in dogs than in humans. This variation is closely related to the dependence of CMA3 binding on the amino acid composition (Hecht et al. 2008), making the significant species‐specific differences in staining behaviour evident and explaining the lower proportion of CMA3‐positive sperm in dogs.

Regarding AcO staining techniques, the study by Warntjen (2012) found that the percentage of sperm with DNA strand breaks was 0.08% in young mice and 0.20% in old mice, while Martins et al. (2007) reported 0.5% in bull sperm. These findings confirm the results obtained in the present study for AcO staining. Contrary to the findings of the current study, Rota et al. (2005) reported values between 2.5% and 6.0% for chromatin‐unstable spermatozoa in dogs. This study indicates that the mean percentage of chromatin‐unstable spermatozoa in dogs is 1.67%. Specifically, fresh sperm showed an average of 1.28% chromatin‐unstable sperm, while cryopreserved samples showed an average of 1.71% AcO‐positive sperm. These findings align with previous studies, supporting the validity of the AcO staining technique. However, it should be noted that Rota et al. (2005) reported higher values for dogs, ranging from 2.5% to 6.0%. Additionally, the analysis of AOT results from 20 cryopreserved semen samples indicated a significant correlation, suggesting that the use of AOT is both reasonable and necessary for assessing chromatin instability in dogs. When comparing different sample types, an increase in acridine orange‐positive (chromatin‐unstable) sperm was observed in cryopreserved samples compared to fresh semen. This observation can partly be explained by the differing subjects in each group and the consistently higher results for cryopreserved samples across various staining methods. Additionally, acridine orange staining specifically indicates DNA integrity.

As mentioned earlier, there are currently few studies that have used the TUNEL test to assess chromatin condensation in dog spermatozoa. The percentages of sperm with fragmented DNA in these studies are significantly lower than the values determined for fertile men. For example, Hammadeh et al. (2010) compared protamination, DNA fragmentation using the TUNEL test, and chromatin condensation using CMA3 in smokers and non‐smokers. They found that the percentage of TUNEL‐positive sperm in 63 non‐smokers was 11.3% ± 4.2%. Similarly, Chohan et al. (2006) reported values of 11.1% ± 0.9% for a group of seven fertile men. An additional study found that TUNEL‐positive sperm accounted for 11.1% in a group of seven fertile men (Chohan et al. 2006). Furthermore, an investigation of frozen–thawed bull sperm samples revealed a much lower percentage of TUNEL‐positive cells, at only 1% (Martins et al. 2007). These findings are consistent with the results observed in the current study. As with previous staining methods, there is no interaction between the sample type and the staining result. This means that the corresponding protocols can be used for both fresh semen and cryopreserved sperm. This is also evident when comparing the respective mean values, which are 0.95% ± 2.07% fragmented DNA for fresh semen and 1.42% ± 1.55% for cryopreserved samples. This staining method also shows a high chromatin instability that canine spermatozoa acquire during their maturation process.

## 5 Conclusion

For veterinary applications without fluorescence microscopy, toluidine blue staining is recommended for its superior contrast and ease of use compared to aniline blue. When fluorescence microscopy is available, CMA3 staining is preferred due to its clarity and cost‐effectiveness, despite health risks, in comparison to TUNEL. Aniline blue, which correlates with TUNEL, offers a rapid and economical method for evaluating chromatin condensation in both fresh and frozen–thawed semen. These findings emphasise the importance of selecting the appropriate staining technique, as significant differences between methods were observed for both fresh and frozen–thawed canine sperm. Toluidine blue is simple but can be time‐consuming and costly, while CMA3 is straightforward, cost‐effective and retains fluorescence over time. Further studies on infertile subjects are needed to assess the practical utility of TUNEL staining in veterinary andrology. For non‐fluorescence microscopy, toluidine blue is recommended, while CMA3 is preferred for fluorescence microscopy, offering clear and economical results.

## Author Contributions

E.‐M.L. was responsible for sampling and analysing the records, with oversight from A.W. and M.E.H. Collaborative efforts of E.‐M.L., A.W., A.F. and M.E.H. were dedicated to critically evaluating the text and statistical results, drafting the manuscript and assessing the sources. The final version of the manuscript was reviewed and approved by all authors.

## Conflicts of Interest

The authors declare no conflicts of interest.

## Data Availability

The data included in this study can be obtained by contacting the corresponding author at afarshad@uok.ac.ir or abbas.farshad@vetmed.unigiessen.de, upon reasonable request.
